# Association of Genetic Ancestry with Breast Cancer in Ethnically Diverse Women from Chicago

**DOI:** 10.1371/journal.pone.0112916

**Published:** 2014-11-25

**Authors:** Umaima Al-Alem, Garth Rauscher, Ebony Shah, Ken Batai, Abeer Mahmoud, Erin Beisner, Abigail Silva, Caryn Peterson, Rick Kittles

**Affiliations:** 1 Division of Epidemiology and Biostatistics, University of Illinois at Chicago, Chicago, Illinois, United States of America; 2 Department of Surgery, Division of Urology, University of Arizona, Tucson, Arizona, United States of America; 3 Department of Pathology, University of Illinois at Chicago, Chicago, Illinois, United States of America; 4 College of Medicine, University of Illinois at Chicago, Chicago, Illinois, United States of America; 5 Edward Hines, Jr. VA Hospital, Hines, Illinois, United States of America; Howard University, United States of America

## Abstract

**Introduction:**

Non-Hispanic (nH) Black and Hispanic women are disproportionately affected by early onset disease, later stage, and with more aggressive, higher grade and ER/PR negative breast cancers. The purpose of this analysis was to examine whether genetic ancestry could account for these variation in breast cancer characteristics, once data were stratified by self-reported race/ethnicity and adjusted for potential confounding by social and behavioral factors.

**Methods:**

We used a panel of 100 ancestry informative markers (AIMs) to estimate individual genetic ancestry in 656 women from the “Breast Cancer Care in Chicago” study, a multi-ethnic cohort of breast cancer patients to examine the association between individual genetic ancestry and breast cancer characteristics. In addition we examined the association of individual AIMs and breast cancer to identify genes/regions that may potentially play a role in breast cancer disease disparities.

**Results:**

As expected, nH Black and Hispanic patients were more likely than nH White patients to be diagnosed at later stages, with higher grade, and with ER/PR negative tumors. Higher European genetic ancestry was protective against later stage at diagnosis (OR 0.7 95%CI: 0.54–0.92) among Hispanic patients, and higher grade (OR 0.73, 95%CI: 0.56–0.95) among nH Black patients. After adjustment for multiple social and behavioral risk factors, the association with later stage remained, while the association with grade was not significant. We also found that the AIM SNP rs10954631 on chromosome 7 was associated with later stage (p = 0.02) and higher grade (p = 0.012) in nH Whites and later stage (p = 0.03) in nH Blacks.

**Conclusion:**

Non-European genetic ancestry was associated with later stage at diagnosis in ethnic minorities. The relation between genetic ancestry and stage at diagnosis may be due to genetic factors and/or unmeasured environmental factors that are overrepresented within certain racial/ethnic groups.

## Introduction

Race and ethnicity are associated with breast cancer incidence and mortality. Black and Hispanic women are more likely than their White counterparts to present at an earlier age, with different breast cancer characteristics such as later stages of breast cancer, and aggressive tumor that have poor prognoses (e.g., negative hormone receptor status, high grade, nuclear atypia, mitotic index, S-phase fraction, and necrosis) [1]–[7]. The causes of the racial and ethnic disparity in breast cancer characteristics are likely a result of both genetic and environmental influences. Several environmental factors experienced during life such as inequalities in health and socioeconomic status have been implicated in the racial disparity in breast cancer prognosis [8]. However, these factors do not completely account for these disparities [9], [10]. It has been postulated that certain biologic characteristics of the tumor might account for at least a portion of the disparity in the breast cancer characteristics and survival [11], [12].

Generally, racial/ethnic groups have been categorized by common geographic origins and shared physical characteristics, such as skin color. Black Americans are primarily a mixture of European and African ancestry, whereas Hispanic Americans are generally a mixture of European, African, and Native American ancestral backgrounds. Racial and ethnic categories used in biomedical research tend to conflate genetic, socioeconomic, social and cultural factors that contribute to racial and ethnic health disparities [12], [13].

Genetic heterogeneity within each racial and ethnic grouping may bias associations in genetic association studies, generating both false-positive and false-negative results [14]–[18]. Variations in the distribution of single nucleotides polymorphisms (SNPs), called ancestry informative markers (AIMs), have been shown to describe the architecture of genome variations between populations [19]. AIMS have been used to test the potential role of genetics in disease disparities within admixed populations [15], [20], [21]. The genetic ancestry proportions assigned to each individual, as opposed to membership of one racial group, serves as a proxy for the genetic ancestral background of the individual and can be used to assess relations between genetic ancestry and breast cancer characteristics. The finding of an association between genetic ancestry and a particular outcome may implicate genetic factors in the differential expression among racial groups.

The relationship between genetic ancestry and breast cancer characteristics has been previously investigated in several studies [20], [22]–[24], but whether genetic ancestry contributes to these differences is still unconfirmed. We use a sociodemographically diverse sample from a population-based study to determine if genetic ancestry, estimated using AIMs, was associated with breast cancer characteristics, after accounting for self-reported race/ethnicity.

## Materials and Methods

### Study population

The study protocol was approved by the University of Illinois at Chicago Institutional Review Board (IRB#2010-0519). All samples were collected with written informed consent from each participant. The University of Illinois at Chicago Institutional Review Board approved the consent forms. Cases were a subset from the parent study “Breast Cancer Care in Chicago” (BCCC). Details of this study have been published elsewhere [25]. Briefly, eligible female patients were between 30 and 79 years of age at diagnosis, resided in Chicago, had a first primary in situ or invasive breast cancer, were diagnosed between October 1, 2005 and February 31, 2008, and self-identified as either non-Hispanic White, non-Hispanic Black or Hispanic. All diagnosing facilities in the greater Chicago area (N = 56) were visited on a monthly basis and all eligible newly diagnosed cases were ascertained. Certified tumor registrars employed by the Illinois State Cancer Registry (ISCR) reviewed pathology records, the hospital tumor registry or both, depending on the protocol at each hospital. Patients were further screened for eligibility and scheduled for interviews if eligible and interested. The 90 minute interview was administered either in English or Spanish-as appropriate- using computer-assisted personal interview procedures. The final interview response rate was 56%, representing 989 completed interviews among eligible patients (397 nH White, 411 nH Black, and 181 Hispanic, and response rates 51%, 59% and 66%, respectively). The interview included questions pertaining to the process of discovery, diagnosis, and treatment of the patient's breast cancer, as well as questions about healthcare-seeking behaviour, sociodemographic background, and known or suspected breast cancer risk factors (i.e., age at menarche, parity, age at first full-term pregnancy, breastfeeding, use of oral contraceptives, use of hormone replacement therapy, family history of breast cancer). Women were asked about the presence of any health problems or existing conditions (i.e., comorbidities) that required seeing a doctor or healthcare practitioner on regular basis at the time of breast cancer diagnosis. Upon completion of the interview, 848 patients consented to allow abstraction of their medical records, including access to their ISCR data and to provide biological samples. Of these, 666 (67%) provided a blood sample.

### Self-reported Race/ethnicity

Self-reported race/ethnicity was defined through separate self-identification of Hispanic ethnicity (yes/no) and race (White/Black). Racial/ethnic groups were categorized as follows: non-Hispanic White (nH White), non-Hispanic Black (nH Black) and Hispanic. Ethnicity was defined as Hispanic if the patient self-identified as Hispanic, reported a Latin American country of origin, or reported a Latin American country of origin for both biological parents.

### Global Genetic Ancestry

DNA from blood was genotyped for 100 AIMs using the Sequenom MassARRAY iPLEX platform. The AIMs panel consisted of carefully selected autosomal markers that were previously identified and validated for estimating continental ancestry information in admixed populations [26]–[28]. All 100 AIMs were genotyped using the Sequenom MassARRAY genotyping platform with iPLEXchemistry according to manufacturer's recommendations. Briefly, iPLEX assays were designed utilizing the Sequenom Assay Design software, allowing for single base extension designs used for multiplexing. PCR and unextended primer sequences may be found within the supplementary materials. Multiplex assays were performed to amplify 5 ng of genomic DNA by polymerase chain reaction (PCR). PCR reactions were treated with shrimp alkaline phosphatase (SAP) to neutralize unincorporated dNTPs. Subsequently, a post-PCR single base extension reaction was performed for each multiplex reaction using concentrations of 0.625 µM for low mass primers and 1.25 µM for high mass primers. Reactions were diluted with 16 µl of H_2_O and fragments were purified with resin, spotted onto Sequenom SpectroCHIP microarrays and scanned by MALDI-TOF mass spectrometry. Individual SNP genotype calls were generated using Sequenom TYPER software, which automatically calls allele specific peaks according to their expected masses. Genotyping quality control for all SNPs was assessed using blinded duplicate genotyping for 60 DNA samples. A genotype concordance rate of 99% was observed for all markers. Genotyping call rates exceeded 98.5% for all individuals included in the analyses.

Individual admixture estimates for each study participant were calculated using a model-based clustering method as implemented in the program STRUCTURE v2.3 [29]. STRUCTURE 2.3 was run using parental population genotypes from west Africans, Europeans, and Native Americans [26] under the admixture model using the Bayesian Markov chain Monte Carlo method (K = 3, assuming three founding populations) and a burn-in length of 30 000 for 70 000 repetitions. Ten cases that self-reported as European American and had more than 70% West African genetic ancestry were excluded. After the exclusions, genotype information was available for a total of 656 cases.

### Statistical analysis

This analysis is based on 656 patients with valid genotyping results (255 NH White, 277 African Americans and 124 Hispanic). Stage at diagnosis, hormone receptor status and histologic grade were abstracted from the patient's medical records. Stage at diagnosis was categorized using the American Joint Committee on Cancer (AJCC) categories of 0,1,2, and 3 and 4. Hormone receptor was defined as positive if tumor contained estrogen (ER) and/or progesterone (PR) receptors, and negative in the absence of both receptor types. Histologic grade was defined as low, intermediate and high. Among the 656 with biological samples, stage at diagnosis was available for 643 cases, histological grade for 575 cases, ER and PR data was available for 600 cases. Later stage at diagnosis was defined as stage 2, 3, 4 vs. 0, 1. Higher grade was defined as grade intermediate and high versus low. ER/PR negative breast cancer was defined as being negative for both ER and PR. As the determination of the Human Epidermal Growth Factor Receptor 2 (Her2) status was not a standard procedure when the BCCC cases were ascertained, we have Her2 status for only 362 cases and only 60 cases with triple negative in our population. Therefore, we excluded Her2 status in the present analysis.

Body mass index (BMI) was calculated as measured weight (kg) divided by measured height (m) squared. Area-level measures of socioeconomic status were based on two well-established measures of neighborhood structural characteristics: concentrated disadvantage and concentrated affluence. The concentrated disadvantage variable was constructed using the following variables derived from the U.S. Census: percent below poverty; percent unemployed; percent receiving public assistance; percent in female-headed households; percent under age 18; and percent African-American [30]. The concentrated affluence variable was constructed using the following Census-derived variables: percent of families with incomes above $75,000; percent of adults with a college education; and percent of the civilian labor force employed in professional or managerial occupations [31].

Baseline characteristics of the population were compared across self-reported racial/ethnic groups using Chi-square statistics tests for categorical variables and ANOVA for continuous variables. Mean genetic ancestry was estimated as the average of the individual genetic ancestry estimates within self-reported racial/ethnic group. We used logistic regression to examine the association between genetic ancestry and breast cancer characteristics within self-reported racial/ethnic group. Genetic ancestry variables were divided equally into fifths at the quintiles within self-reported racial ethnic groups. Separate models were run for each self-reported racial/ethnic group (nH White, nH Black and Hispanic), ancestry (European, West-African, and Native American) and tumor characteristic (later stage, higher grade, ER/PR negative) to estimate the odds ratio and 95% confidence interval for the highest to the lowest fifth of genetic ancestry. The choice of quintiles was based on the assumption that if there was an effect of ancestry it was likely to be monotonic such that the effect would increase with increasing ancestry. We performed several other categorizations for modeling in ordinal logistic regression and they gave similar results. The regression models were adjusted for health insurance, income, education, concentrated disadvantage, concentrated affluence, nulliparity, and age at first and last birth. All reported p-values are two-sided. Statistical analyses were conducted using Stata version 11 (College Station, TX). The association between SNPs in our AIMs panel (Table S1) and breast cancer characteristics were tested using PLINK after removing individuals with >5% missing genotypes and adjusting for corresponding genetic ancestry category, health insurance, income, education, disadvantage, affluence, nulliparity, and age at first and last birth. We run logistic regression models stratified by self-reported race/ethnicity adjusting for genetic ancestry. For nH Black cases, we adjusted for West African Ancestry and for Hispanic cases we adjusted for both West African Ancestry and Native American ancestry. We did not need to adjust for European genetics ancestry among nH White cases.

## Results

### Baseline characteristics of the cohort

The tumor and demographic characteristics of the final cohort which includes a total of 250 White, 273 Black, and 120 Hispanic women are summarized in Table 1. The mean age at diagnosis was 55 years (range 25 to 78 years). Racial/ethnic disparities in breast cancer characteristics were apparent in this population, as nH Black and Hispanic women were diagnosed at a later stage, higher grade and with a higher proportion of ER/PR negative tumors, compared to nH Whites. In addition, a greater proportion of nH Black and Hispanic women were overweight/obese, had more co-morbidities, were less likely to have their cancer detected through screening mammography, had a lower level of education and income, and less likely to have private insurance than nH Whites. The distribution of tumor characteristics and breast cancer risk factors of this subset was similar to the full cohort (Table S2).

**Table 1:** Descriptive and tumor characteristics of the BCCC sample stratified by self-reported race/ethnicity.

	Total	nH White	nH Black	Hispanic	p-value
	n	%	%	%	
**Age, mean(±SD)**	656	55(11)	56(11)	53(11)	0.138
**Age at first birth, mean(±SD)**	656	26(±6)	21(±5)	23(±6)	<0.0001
**Age at last birth, mean(±SD)**	656	31(±6)	29(±6)	31(±6)	<0.0001
**Stage at diagnosis (n = 643)**					
0,1 (early stage)	374	67	55	48	0.0004
2,3,4 (late stage)	269	33	45	52	
**Histologic grade (n = 575)**					
Low/intermediate	348	67	55	61	0.025
High	227	34	45	39	
**ER/PR status (n = 600)**					
ER and/or PR Positive	474	86	55	61	<0.001
Double negative	126	14	45	39	
**Her2 overexpression (n = 362)**					
No	305	90	78	86	0.028
Yes	57	10	22	14	
**Body mass index (kg/m^2^) (n = 652)**					
Normal weight (18.5–24.9)	202	49	20	20	<0.0001
Overweight (25.0–29.9)	194	22	29	48	
Obese (≥30.0)	256	29	52	32	
**Any co-morbidities (n = 656)**					
No	286	49	37	48	0.007
Yes	370	51	63	52	
**Nulliparity (n = 656)**					
Yes	133	37	10	7	0.007
No	523	63	90	93	
**Menopausal status (n = 649)**					
No	132	17	20	27	0.105
Yes	517	83	80	73	
**Mode of Breast cancer detection (n = 656)**					
Screen detected	336	60	45	45	0.003
Symptomatic	320	40	55	55	
**Education (n = 655)**					
less than High school	120	4	20	44	<0.0001
High school	138	15	27	21	
some college	397	81	53	35	
**Annual household Income (n = 655)**					
less than $30,000	263	17	56	57	<0.0001
$30,000 to $75,000	277	52	38	37	
Greater than $75,000	102	31	6	7	
**Insurance category (n = 656)**					
No outpatient insurance	84	7	14	23	<0.0001
Public	125	4	31	23	
Private	447	89	55	55	

P-values for categorical variables are from χ^2^ tests and from ANOVA for continuous variables for differences according to self-reported race/ethnicity.

**Figure 1:**
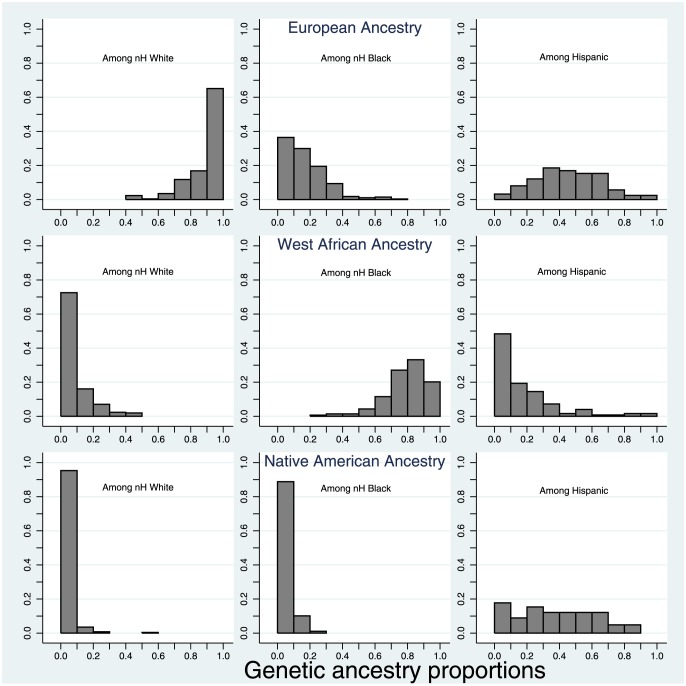
The distribution of European ancestry, West African ancestry, and Native American genetic ancestry stratified on self-reported race/ethnicity.

The distribution of estimated West African, European and Native American ancestry varied among the three self-reported racial groups (Figure 1). The predominant genetic ancestry proportion among White cases was the European genetic ancestry, with a mean of 90% (±SD 11%). The predominant genetic ancestry among Black cases was West African genetic ancestry, with a mean of 79% (±SD 13%). Hispanic women had a wide range of European (mean 45%), Native American (mean 37%) and West African (mean 18%) genetic ancestry representing a highly admixed group.

### Genetic ancestry and Breast cancer:

We examined the association between genetic ancestry (modeled as an ordinal variable in fifths) and tumor characteristics within self-reported racial/ethnic groups (Table 2). Greater European ancestry (top quintile versus lowest quintile) was protective against late stage (Hispanics: OR 0.70, 95%, 0.54–0.92; nH Blacks: OR 0.88, 95%CI: 0.75–1.05). Among Hispanics it was also protective against higher grade (OR 0.73, 95% CI: 0.56–0.95%). On the other hand, greater West-African ancestry was associated with higher grade (OR 1.36, 95%CI: 1.05–1.77) and later stage (OR 1.13, 95%CI: 0.95–1.34) among nH Blacks. Native American ancestry was associated with later stage at diagnosis (OR 1.36, 95%CI: 1.04–1.79).

**Table 2:** Relations between genetic ancestry and breast cancer characteristics within racial/ethnic subgroups. Genetic ancestry divided into fifths within each subgroup.

		Unadjusted	Adjusted^a^
Ancestry/Subgroup	n	OR (95% CI)	OR (95% CI)
**Later Stage at Diagnosis**			
**European Ancestry**			
Among nH Whites	250	0.97 (0.80–1.17)	1.13 (0.88–1.47)
Among nH Blacks	273	0.88 (0.75–1.05)+	0.84 (0.70–1.02)*
Among Hispanics	120	0.70 (0.54–0.92)***	0.58 (0.41–0.80)***
**West African Ancestry**			
Among nH Blacks	273	1.13 (0.95–1.34)+	1.15(0.97–1.41)*
**Native American Ancestry**			
Among Hispanics	120	1.36 (1.04–1.79)**	1.35 (1–1.83)**
**Higher Grade Disease**			
**European Ancestry**			
Among nH Whites	224	1.06 (0.86–1.31)	1.05 (0.79–1.41)
Among nH Blacks	242	0.73 (0.56–0.95)**	0.79(0.6–1.05)*
Among Hispanics	109	1.04 (0.69–1.56)	0.95 (0.57–1.56)
**West African Ancestry**			
Among nH Blacks	242	1.36 (1.05–1.77)**	1.26 (0.95–1.67)*
**Native American Ancestry**			
Among Hispanics	109	1.07 (0.70–1.62)	1.23 (0.73–2.06)
**ER/PR Negative Status**			
**European Ancestry**			
Among nH Whites	228	1.18 (0.90–1.56)	1.20 (0.81–1.78)
Among nH Blacks	240	0.97 (0.82–1.18)	1.04 (0.84–1.29)
Among Hispanics	110	1.13 (0.81–1.56)	1.05 (0.75–1.49)
**West African Ancestry**			
Among nH Blacks	240	0.84 (0.65–1.10)	1.03 (0.83–1.28)
**Native American Ancestry**			
Among Hispanics	110	1.04 (0.74–1.46)	1.10 (0.76–1.60)

+ p<0.20,

*p<0.10,

**p<0.05,

***p<0.01.

OR, odds ratio from logistic regression comparing the highest versus the lowest fifth of the subsample distribution.

a Adjusted for health insurance, income, education, disadvantage, affluence, nulliparity, and age at first and last birth.

The associations between genetic ancestry and breast cancer characteristics were generally not attenuated after adjustment for social and behavioral factors as the unadjusted and adjusted point estimates were similar, but CI widened and lost statistical significance at p<0.05 (Table 2).

We also examined the association of individual SNPs in our AIMs panel (Table S2) and breast cancer characteristics among our three self-reported racial/ethnic categories. In unadjusted model, two AIMs were significantly associated in nH Whites after Bonferroni correction (p<0.0005). SNPs rs11073967 on chromosome 15 was associated with later stage (p = 9.9×10^−5^), and rs10954631 on chromosome 7 was associated with ER/PR negative status (p = 2.6×10^−4^). However, only rs10954631 remained statistically significant after adjusting for social and behavioral factors (p = 1.1×10^−4^). The rs10954631 SNP was also associated with other breast cancer characteristics: later stage (p = 0.02) and higher grade (p = 0.012) in nH White as well as with later stage (p = 0.03) in nH Black. Data summarised in Table S3 (Stage at diagnosis), Table S4 (Grade at diagnosis) and Table S5 (ER_PR positivity).

## Discussion

We used ancestry informative markers to estimate individual genetic ancestry in a multi-racial cohort of breast cancer patients and examined the association between genetic ancestry and breast cancer characteristics after accounting for self-reported race/ethnicity.

In our population of incident breast cancer patients, nH Whites and Blacks had 79% European and 90% West African ancestries respectively. Hispanics, however, had a broader heterogeneous mixture of West African, European, and Native American ancestries. While this ethnic category represents individuals who, for most part, share a common language, it actually encompasses groups of individuals that differ in terms of their genetic ancestry proportions [32], [33]. The average West African ancestry we estimated in our sample of Hispanics was 18%, which is much higher than that observed in Mexicans and Mexican Americans (4–7%) [20], [34], and is more similar to Puerto Ricans (21%) [35].

When assessing the relationship between genetic ancestry and breast cancer characteristics, we found that Native American genetic ancestry was associated with later stage at diagnosis and West African genetic ancestry was associated with higher grade in ethnic minorities. These associations could not be explained by multiple social and behavioral risk factors. The finding that genetic ancestry is associated with stage of breast cancer suggests that genetic factors may play a role in the observed breast cancer disparities. Alternatively, this could be due, in part to the strong correlation between genetic ancestry and self-reported race/ethnicity.

Our results are consistent with Fejerman et al. [22] and Palmer et al [23] who also found that genetic ancestry was associated with stage at diagnosis among nH Blacks. Similar to Reding et al [24], but in contrast to Fejerman et al and Palmer et al, we did not find an association between ER/PR status and West African genetic ancestry among nH Blacks. Our sample size may have limited our ability to detect associations between ancestry and ER/PR status in nH Blacks. However, we did observe several associations between ancestry and breast cancer characteristics that have not been previously reported. For instance, higher West African ancestry increased the odds of higher grade among nH Blacks. However, after adjustment for multiple social and behavioral risk factors, the point estimate was similar to unadjusted models but lost statistical significance at p = 0.05. We also observed an association between Native American ancestry and stage at diagnosis that was not previously seen in Hispanic women from the US or Mexico. Our results differ from those of Fejerman and colleagues [20] who analyzed the effect of ancestry on stage, grade and other tumor characteristics in U.S. Latinas and did not find any statistically significant associations. The differences between our study and those of Fejerman et al. [20] could be attributed to variation in the case populations. The Fejerman et al study consisted of Hispanic women from the San Francisco Bay area while our study in the Chicago metropolitan area contained a larger proportion of Hispanic women from the Caribbean. As previously stated, the proportion of Native American and West African genetic ancestry is significantly different between Hispanics of Mexican origin and those from the Caribbean, thus genetic ancestry in each group might be a proxy for a different risk factor. This highlights why investigators should not generalize findings across all Hispanic populations.

We examined the association of individual AIMs markers with breast cancer characteristics to identify whether these markers are in linkage disequilibrium with regions that may potentially play a role in breast cancer disease disparities. We found that the SNP rs10954631 on chromosome 7 was associated with later stage (p = 0.02) and higher grade (p = 0.012) in nH White women as well as with later stage (p = 0.03) in nH Black women.

SNP rs10954631 is located in the *KIAA1549* gene which is about 2 mb downstream of the BRAF oncogene. The KIAA1549 gene is often fused to the BRAF in cases of pilocytic astrocytoma [36]. This SNP is located in an interesting region of chromosome 7 as several associations with breast cancer were observed. The 7q34 region on chromosome 7 was shown to be associated with lobular breast cancer specific predisposition [37]. SNP rs10954631 is located about 500 kb downstream of Transcription intermediary factor 1α (TIF-1α) gene -also known as TRIM24. Overexpression of the TRIM24/IF-1 gene in breast cancer is associated with poor prognosis and worse survival [38]. Gross Cystic Disease Fluid Protein-15(GCDFP-15)/Prolactin-Inducible Protein (PIP) expression is associated with invasive breast cancer [39]. There are also several genes close to this region that are associated with many types of cancer including breast cancer, EPH receptor B6 (EPHB) [40], [41] and Transient receptor potential vanilloid 6 (TRPV6) [42]. Further analysis of this region is needed.

A major strength of this study is the use of a sociodemographically diverse sample that capture the three major racial/ethnic groups from a population-based study to assess the relationship between genetic ancestry and breast cancer. Nonetheless there are limitations to these analyses. In addition to the relatively small sample size, potential misclassification of ER/PR status, grade and stage might tend to alter observed associations in unpredictable ways, by either attenuating or biasing associations away from the null. Finally, it is important to emphasize that the association of stage at diagnosis with genetic ancestry does not necessarily represent racially distributed genetic factors. It was not possible to adjust completely for all the myriad ways in which social, behavioral, and health care access differences that could have contributed to tumor characteristics, and as such we cannot interpret the adjusted relations of genetic ancestry with tumor characteristics as being the sole result of ancestral origin. Nonetheless, our inability to adjust away these associations by including multiple social and health care access variables in our models leaves open the possibility that differences in genetic ancestry contribute to racial/ethnic disparities in tumor characteristics.

### Conclusion

Differences in breast cancer aggression among different racial and ethnic groups have been previously reported, but whether genetic ancestry contributes to these differences remain unknown. Our study reveals that genetic ancestry plays a role in breast cancer. We used diverse samples from a population-based study and found that non-European genetic ancestry was associated with later stage and grade at diagnosis in ethnic minorities. Future studies investigating the relation between genetic ancestry and stage at diagnosis are warranted. As this relationship may be due to genetic factors and/or unmeasured environmental factors that are overrepresented within certain racial/ethnic groups.

## Supporting Information

Table S1 **Ancestry Informative Marker Panel.**
(DOCX)

Table S2 **Descriptive and tumor characteristics of the full BCCC cohort stratified by self-reported race/ethnicity.**
(DOCX)

Table S3 **Table S3 Association of Grade at diagnosis and ancestry.**
(XLSX)

Table S4 **Association of Stage at diagnosis and ancestry.**
(XLSX)

Table S5 **Association of ER_PR status and ancestry.**
(XLSX)
