# Supplementary material for: Association of Genetic Ancestry with Breast Cancer in Ethnically Diverse Women from Chicago
Source: PLoS One. 2014 Nov 25;9(11):e112916. doi: 10.1371/journal.pone.0112916 (PMC4244099; doi:10.1371/journal.pone.0112916)
Supplement: Table S1 — Ancestry Informative Marker Panel. (DOCX) [file pone.0112916.s001.docx]

| **Chromosome** | **SNP** | **Physical Position (BP)** | **nH White** | **nH Black** | **Hispanic** |
| --- | --- | --- | --- | --- | --- |
| 1 | rs424436 | 8082678 | C | C | C |
| 1 | rs7504 | 27238150 | A | A | G |
| 1 | rs1931059 | 35366056 | A | A | A |
| 1 | rs10908316 | 35494810 | G | T | G |
| 1 | rs2791966 | 36314861 | T | C | C |
| 1 | rs710232 | 42203154 | C | T | C |
| 1 | rs596985 | 64277035 | C | T | C |
| 1 | rs855833 | 64357432 | C | T | C |
| 1 | rs17035850 | 117032022 | T | A | T |
| 1 | rs5025718 | 120478285 | T | C | T |
| 1 | rs6695965 | 147060091 | C | T | T |
| 1 | rs2274533 | 151395782 | T | T | T |
| 1 | rs2814778 | 159174683 | G | A | G |
| 1 | rs12074150 | 173616907 | G | G | G |
| 1 | rs2065160 | 204790977 | C | C | C |
| 1 | rs6604611 | 216655251 | A | C | A |
| 1 | rs6698938 | 229985356 | G | G | G |
| 1 | rs2502342 | 243070467 | T | C | T |
| 2 | rs883399 | 9634100 | G | A | A |
| 2 | rs300152 | 17986683 | T | T | T |
| 2 | rs2384319 | 26206255 | G | G | G |
| 2 | rs11124405 | 35188011 | G | A | G |
| 2 | rs13385952 | 41531398 | C | T | C |
| 2 | rs3768641 | 72368190 | C | G | C |
| 2 | rs1881244 | 73645089 | A | G | A |
| 2 | rs12714168 | 86331347 | C | T | C |
| 2 | rs6576989 | 97525099 | C | T | C |
| 2 | rs260714 | 109562495 | T | C | C |
| 2 | rs951954 | 110459505 | A | G | A |
| 2 | rs901304 | 163416362 | C | T | C |
| 2 | rs6748661 | 195682840 | A | G | A |
| 3 | rs11713766 | 398089 | A | G | A |
| 3 | rs2470644 | 5776857 | G | A | G |
| 3 | rs2197896 | 30032530 | C | T | C |
| 3 | rs9311121 | 35928063 | T | C | T |
| 3 | rs13069719 | 71506545 | T | T | T |
| 3 | rs2660769 | 87080496 | G | A | G |
| 3 | rs12489482 | 104579408 | A | G | A |
| 3 | rs6437783 | 108172817 | C | C | C |
| 3 | rs11714866 | 110067902 | G | A | A |
| 3 | rs6772085 | 118584563 | C | T | C |
| 3 | rs2165139 | 139214470 | T | T | T |
| 3 | rs6439896 | 139864353 | C | T | C |
| 3 | rs1439013 | 152581991 | C | T | C |
| 3 | rs9290363 | 168995107 | G | T | G |
| 4 | rs10032047 | 63727833 | A | G | A |
| 4 | rs7689609 | 72083374 | C | T | C |
| 4 | rs6446975 | 75036044 | A | G | A |
| 4 | rs7687935 | 82065566 | T | A | T |
| 4 | rs7662047 | 103091730 | A | G | A |
| 4 | rs7657799 | 105375423 | G | T | G |
| 4 | rs13108157 | 151530763 | T | C | T |
| 4 | rs6829588 | 165221971 | T | G | T |
| 4 | rs2332031 | 171742958 | C | T | C |
| 5 | rs814597 | 10468929 | T | T | T |
| 5 | rs463240 | 25845146 | A | G | A |
| 5 | rs35395 | 33948589 | T | C | C |
| 5 | rs16891982 | 33951693 | C | G | G |
| 5 | rs10059859 | 59231307 | T | C | T |
| 5 | rs1443985 | 119425507 | G | A | G |
| 5 | rs4513684 | 147652085 | C | A | C |
| 5 | rs1551765 | 153176578 | T | T | T |
| 6 | rs6909271 | 198379 | A | T | A |
| 6 | rs6459548 | 17482317 | A | G | A |
| 6 | rs2497150 | 84847013 | T | C | T |
| 6 | rs794672 | 95458317 | A | G | A |
| 6 | rs218867 | 121398535 | G | A | A |
| 7 | rs7784684 | 40167762 | T | G | T |
| 7 | rs10264353 | 43321077 | G | A | G |
| 7 | rs10257477 | 107704688 | C | T | C |
| 7 | rs4727700 | 107807083 | T | A | T |
| 7 | rs3094537 | 109554669 | C | T | C |
| 7 | rs2021782 | 132134995 | G | A | G |
| 7 | rs10954631 | 138539626 | A | G | A |
| 8 | rs6601288 | 8943430 | A | A | T |
| 8 | rs11778591 | 12720349 | C | C | A |
| 8 | rs2439522 | 97533766 | G | G | G |
| 8 | rs1871534 | 145639681 | G | C | G |
| 9 | rs12347078 | 344508 | C | A | C |
| 9 | rs4478653 | 21853221 | C | T | T |
| 9 | rs587364 | 125760863 | T | C | T |
| 10 | rs10748592 | 94878355 | T | T | G |
| 11 | rs6485600 | 12259702 | A | G | G |
| 11 | rs1638567 | 67125223 | C | T | C |
| 11 | rs2458640 | 78035856 | C | A | A |
| 11 | rs533571 | 100850202 | A | A | G |
| 11 | rs4936512 | 120155336 | C | C | T |
| 11 | rs1648180 | 128049402 | A | A | G |
| 12 | rs2293048 | 117664825 | T | T | T |
| 13 | rs2065982 | 34864240 | C | C | C |
| 13 | rs1540979 | 95090692 | T | T | T |
| 14 | rs730570 | 101142890 | G | A | A |
| 15 | rs2714758 | 25479337 | G | A | G |
| 15 | rs1129038 | 28356859 | G | A | A |
| 15 | rs1426654 | 48426484 | G | A | G |
| 15 | rs11073967 | 91565804 | G | A | A |
| 16 | rs9937955 | 10950526 | A | A | A |
| 16 | rs1557519 | 14251303 | C | T | C |
| 23 | rs992864 | 33796891 | A | G | A |
| 23 | rs1867024 | 147980413 | G | A | G |
