# Supplementary material for: Association of Genetic Ancestry with Breast Cancer in Ethnically Diverse Women from Chicago
Source: PLoS One. 2014 Nov 25;9(11):e112916. doi: 10.1371/journal.pone.0112916 (PMC4244099; doi:10.1371/journal.pone.0112916)
Supplement: Table S2 — Descriptive and tumor characteristics of the full BCCC cohort stratified by self-reported race/ethnicity. (DOCX) [file pone.0112916.s002.docx]

**Supplementary Table 2.** Descriptive and tumor characteristics of the full BCCC cohort stratified by self-reported race/ethnicity.

|  | **Total** | **nH Whites** | **nH Blacks** | **Hispanics** | **p-value** |
| --- | --- | --- | --- | --- | --- |
|  | n | % | % | % |  |
| **Age, mean (±SD)** | 656 | 56(11) | 56(11) | 53(11) | 0.09 |
| **Age at first birth, mean(±SD)** | 656 | 26(6) | 21(5) | 24(6) | <0.0001 |
| **Age at last birth mean(±SD)** | 656 | 31(6) | 28(6) | 32(6) | <0.0001 |
| **Stage at diagnosis (n= 850 )** |  |  |  |  |  |
| 0,1 (early stage) | 487 | 65 | 55 | 48 | 0.001 |
| 2,3,4 (late stage) | 363 | 35 | 45 | 52 |  |
| **Histologic grade (n= 839)** |  |  |  |  |  |
| Low/intermediate | 543 | 69 | 60 | 65 | 0.052 |
| High | 296 | 31 | 40 | 35 |  |
| **ER/PR status (n= 774)** |  |  |  |  |  |
| ER and/or PR Positive | 611 | 86 | 72 | 79 | <0.0001 |
| Double negative | 163 | 14 | 28 | 21 |  |
| **Her2/neu overexpression (n=479)** |  |  |  |  |  |
| No | 395 | 87 | 78 | 82 | 0.046 |
| Yes | 84 | 13 | 22 | 18 |  |
| **Body mass index (kg/m^2^) (n= 983)** |  |  |  |  |  |
| Normal weight (18.5 – 24.9) | 321 | 51 | 20 | 23 | <0.0001 |
| Overweight (25.0 – 29.9) | 307 | 24 | 32 | 44 |  |
| Obese (≥30.0) | 355 | 25 | 48 | 33 |  |
| **Any co-morbidities (n=989)** |  |  |  |  |  |
| No | 446 | 51 | 38 | 49 | <0.0001 |
| Yes | 543 | 59 | 62 | 50 |  |
| **Nulliparity (n=989)** |  |  |  |  |  |
| Yes | 216 | 39 | 11 | 9 | <0.0001 |
| No | 773 | 61 | 89 | 91 |  |
| **Menopausal status (n=981)** |  |  |  |  |  |
| No | 192 | 18 | 18 | 27 | 0.029 |
| Yes | 789 | 82 | 82 | 73 |  |
| **Mode of Breast cancer detection (n= 989)** | |  |  |  |  |
| Screen detected | 507 | 59 | 47 | 42 | <0.0001 |
| Symptomatic | 482 | 41 | 53 | 58 |  |
| **Education (n= 986)** |  |  |  |  |  |
| less than High school | 176 | 5 | 19 | 44 | <0.0001 |
| High school | 193 | 13 | 25 | 21 |  |
| some college | 620 | 82 | 56 | 35 |  |
| **Annual household Income (n=960)** |  |  |  |  |  |
| less than $30,000 | 365 | 16 | 52 | 54 | <0.0001 |
| $30,000 to $75,000 | 336 | 35 | 35 | 34 |  |
| Greater than $75,000 | 259 | 49 | 13 | 12 |  |
| **Insurance category (n= 989)** |  |  |  |  |  |
| No outpatient insurance | 128 | 6 | 15 | 25 | <0.0001 |
| Public | 164 | 4 | 26 | 22 |  |
| Private | 697 | 90 | 59 | 53 |  |
| P-values for categorical variables are from χ^2^ tests and from ANOVA for continuous variables for differences according to self-reported race/ethnicity | | | | | |
